# *In Vivo* Protein Interactions and Complex Formation in the *Pectobacterium atrosepticum* Subtype I-F CRISPR/Cas System

**DOI:** 10.1371/journal.pone.0049549

**Published:** 2012-12-03

**Authors:** Corinna Richter, Tamzin Gristwood, James S. Clulow, Peter C. Fineran

**Affiliations:** Department of Microbiology and Immunology, University of Otago, Dunedin, New Zealand; University of the West of England, United Kingdom

## Abstract

Clustered Regularly Interspaced Short Palindromic Repeats (CRISPR) and their associated proteins (Cas; CRISPR associated) are a bacterial defense mechanism against extra-chromosomal elements. CRISPR/Cas systems are distinct from other known defense mechanisms insofar as they provide acquired and heritable immunity. Resistance is accomplished in multiple stages in which the Cas proteins provide the enzymatic machinery. Importantly, subtype-specific proteins have been shown to form complexes in combination with small RNAs, which enable sequence-specific targeting of foreign nucleic acids. We used *Pectobacterium atrosepticum*, a plant pathogen that causes soft-rot and blackleg disease in potato, to investigate protein-protein interactions and complex formation in the subtype I-F CRISPR/Cas system. The *P. atrosepticum* CRISPR/Cas system encodes six proteins: Cas1, Cas3, and the four subtype specific proteins Csy1, Csy2, Csy3 and Cas6f (Csy4). Using co-purification followed by mass spectrometry as well as directed co-immunoprecipitation we have demonstrated complex formation by the Csy1-3 and Cas6f proteins, and determined details about the architecture of that complex. Cas3 was also shown to co-purify all four subtype-specific proteins, consistent with its role in targeting. Furthermore, our results show that the subtype I-F Cas1 and Cas3 (a Cas2-Cas3 hybrid) proteins interact, suggesting a protein complex for adaptation and a role for subtype I-F Cas3 proteins in both the adaptation and interference steps of the CRISPR/Cas mechanism.

## Introduction

Despite the ability of bacteriophages and plasmids to positively contribute to the rapid evolution of bacteria, these interactions are not always favourable. For example, infection with lytic phages typically results in the death of host bacteria, whereas plasmids can be a fitness burden when their cost outweighs any adaptive advantage conferred [1]. Therefore, it is not surprising that bacteria have developed multiple mechanisms to resist mobile genetic elements [2], [3]. In recent years particular attention has been focussed on the Clustered Regularly Interspaced Short Palindromic Repeat (CRISPR) systems and their CRISPR associated (Cas) genes. CRISPR/Cas systems provide an acquired immunity against both phage and plasmids [4], [5]. These systems are comprised of one or more CRISPR arrays with upstream leader sequences and closely-associated *cas* genes, which encode the proteins required for resistance [6], [7]. CRISPR arrays contain unique sequences, termed spacers, which are derived from phage or plasmid “protospacer” sequences. It is these spacer sequences that provide the resistance specificity [8], [9]. CRISPR arrays are then transcribed and processed to generate short crRNAs (CRISPR RNAs) that, in combination with Cas proteins, target and degrade invading genetic material [10], [11].

There is significant variation in CRISPR/Cas systems [7], [12], which recently led to their reclassification [13], [14]. The major types, I – III, are distinguished based upon signature proteins, which are Cas3, Cas9, and Cas10 for type I, II, and III, respectively. The major types comprise further subtypes (e.g. I-A to I-F), each characterized by a specific set of proteins [13], [14]. The functional mechanism of CRISPR/Cas consists of three stages: 1) acquisition of resistance, 2) CRISPR RNA biogenesis and 3) interference [15]. During acquisition, new spacers derived from the protospacer sequence of the invading phage or plasmid are incorporated into the CRISPR array. Incorporation typically occurs at the end proximal to the leader [16], [17] and, as such, forms a chronological record of past invasions. The leader contains the promoter for CRISPR expression [18]–[20]. In contrast, a recent study in *Sulfolobus solfataricus* showed that some CRISPR arrays utilise an internal spacer incorporation mechanism [21]. Acquisition requires Cas1 and Cas2 [17], [22], which are the only two proteins conserved across all subtypes [14]. Sequences adjacent to the protospacers (termed protospacer adjacent motifs (PAMs)) [8] are important for incorporation of new spacers [17].

During CRISPR RNA biogenesis and interference, the CRISPR array is transcribed into one long pre-crRNA, which is cleaved into small mature crRNAs that consist of remnants of the repeat and an entire or truncated spacer also referred to as guide sequence [11], [23]–[25]. Cas6, Cas6e (CasE/Cse3) and Cas6f (also known as Csy4) have been identified as the endoribonucleases in *Pyrococcus furiosus* (subtype III-B), *Staphylococcus epidermidis* (subtype III-A), *Escherichia coli* (subtype I-E) and *Pseudomonas aeruginosa* and *Pectobacterium atrosepticum* (both subtype I-F) [10], [20], [24], [26], [27]. The mature crRNAs guide a complex of subtype-specific Cas proteins to the invading nucleic acid, which is subsequently degraded. Formation of such protein-RNA complexes has been shown for *E. coli* (subtype I-E) [10], [28]–[30], *P. furiosus* and *Sulfolobus solfataricus* (both subtype III-B) [11], [25], [31], *Streptococcus pyogenes* (subtype II-A) [32], *S. solfataricus* (subtype I-A) [33], *Bacillus halodurans* (subtype I-C) [34] and recently *P. aeruginosa* (subtype I-F) [35]. The CRISPR-associated complex for antiviral defense (Cascade) from *E. coli* is the most well characterised system [28], [29]. The *E. coli* Cascade complex that contains mature crRNA recognises target DNA and recruits Cas3, a protein with both nuclease and helicase activity [36]–[38] that is required for interference [10] by degrading invader DNA [38].

**Figure 1:**
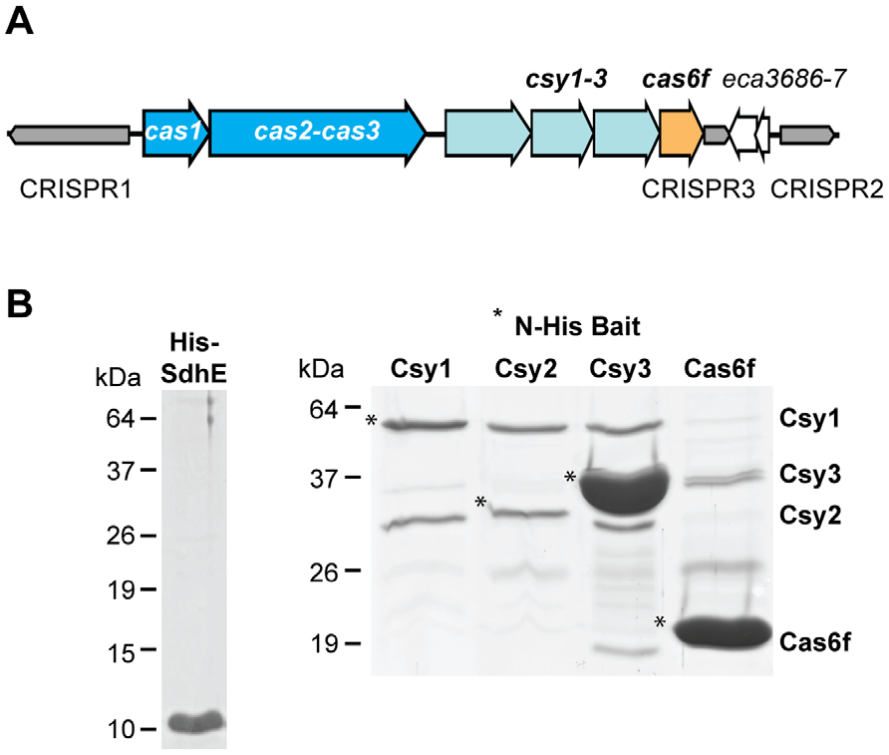
The Csy1-3 and Cas6f proteins of *P. atrosepticum* form a complex *in vivo*. (A) Scale schematic representation of the CRISPR/Cas system in *P. atrosepticum* strain SCRI1043. The 3 CRISPR loci are denoted CRISPR1-3 in order of decreasing length and the direction of transcription indicated by the directionality of the arrows. The universal and type-specific genes, *cas1* and *cas2-cas3* are shown in blue and the subtype I-F-specific genes are depicted in light blue (*csy1-3*) and orange (*cas6f*). Between CRISPR2 and CRISPR3 is a putative toxin-antitoxin system (*eca3686-7*). (B) Co-purification of Csy1-3 and Cas6f proteins using Ni-NTA agarose. Coomassie stained SDS-PAGE gel of elution fractions from the *P. atrosepticum* Δ*cas* mutants expressing untagged Csy1-3 and Cas6f (pJSC11) and either one of the four different His-tagged bait Csy and Cas6f proteins (plasmids pJSC3-6 encode His-tagged Csy1-3 and Cas6f, respectively) or an His-tagged SdhE control (pMAT4). Proteins were identified by MS as indicated and results also shown in Table 3.

*Pectobacterium atrosepticum* (formerly *Erwinia carotovora* subsp. *atroseptica*) is an economically important γ-proteobacterial plant pathogen that causes soft-rot and blackleg disease in potato [39]. Previously, we demonstrated that *P. atrosepticum* strain SCRI1043 contains a subtype I-F CRISPR/Cas system with *cas1, cas3, csy1-3* and *cas6f* and three CRISPR arrays (Fig. 1A) [20]. These arrays and the *cas* genes are transcribed under laboratory conditions, and the CRISPR RNAs are processed both *in vivo* and *in vitro* by the endoribonuclease Cas6f [20]. The CRISPR repeats in all three arrays are 28 nt long with a consensus sequence of GTTCACTGCCGTACAGGCAGCTTAGAAA and are interspersed with 32 nt spacers. CRISPR1-3 possess 28, 10 and 3 spacers, respectively with no homology to known phages or plasmid sequences, yet spacer 6 in CRISPR2 shows 100% identity to a region in *eca0560* within its own genome [20]. CRISPR2 and 3 are separated by a hypothetical toxin-antitoxin system (*eca3686-7*).

Here, we investigated complex formation and pairwise protein interactions in the CRISPR/Cas subtype I-F system. We report formation of a *P. atrosepticum* Csy1-3 and Cas6f complex (referred to as the Csy complex for simplicity), complementing the results published for a related system in *P. aeruginosa* [35]. We have further probed the *in vivo* architecture of the complex by analysing individual protein-protein interactions in wild-type and *cas* deletion backgrounds. In addition, we provide the first evidence that subtype I-F Cas3 interacts with Cas1 and the Csy complex, which may have implications for both the integration of new spacers and the interference mechanism.

## Materials and Methods

### Bacterial Strains and Growth Conditions

*Pectobacterium atrosepticum* strain SCRI1043 [39] wild type (WT) and PCF80 (Δ*cas*::*cat*) [20] strains were grown at 25°C and *E. coli* DH5α at 37°C in Luria Broth (LB) at 200 rpm or on LB-agar plates containing 1.5% (w/v) agar. Bacterial growth (OD_600_) and absorbance were measured in a Jenway 6300 spectrophotometer. When required, medium was supplemented with the following antibiotics: ampicillin (100 µg/ml), and kanamycin (50 µg/ml).

### Oligonucleotides, Cloning, Plasmids and Sequencing

Molecular biology methods were performed using standard techniques. PCR was performed using Phusion DNA polymerase (Finnzymes) for cloning, or Taq polymerase (Roche) for colony screening. PCR products and digested plasmid DNA were purified using the Illustra™ GFX™ PCR DNA and gel band purification kit. Ligations were performed using NEB or Roche T4 ligase. Plasmid DNA was purified using Qiagen™ DNA purification kit and Zippy™ DNA purification kits following the manufacturers’ instructions. All plasmids used in this study were confirmed by sequencing and are listed in Table 1 and oligonucleotides are listed in Table 2. DNA sequencing was performed at the DNA sequencing facility, Allan Wilson Centre, Massey University, New Zealand. Nucleotide sequence data was analysed using Chromas Lite.

**Table 1:** Plasmids used in this study.

Plasmid	Details	Reference
pBAD30	Bacterial expression vector, p15A/M13 replicon, Ap^R^	[54]
pCR19	N-term FLAG-tagged Csy2, pBAD30 derivative, Ap^R^	This study
pCR20	C-term FLAG-tagged Csy3, pBAD30 derivative, Ap^R^	This study
pCR21	N-term FLAG-tagged Csy3, pBAD30 derivative, Ap^R^	This study
pCR22	C-term FLAG-tagged Csy2, pBAD30 derivative, Ap^R^	This study
pJSC1	N-term His-tagged Cas1, pTRB30 derivative, Km^R^	[20]
pJSC2	N-term His-tagged Cas3, pTRB30 derivative, Km^R^	[20]
pJSC3	N-term His-tagged Csy1, pTRB30 derivative, Km^R^	[20]
pJSC4	N-term His-tagged Csy2, pTRB30 derivative, Km^R^	[20]
pJSC5	N-term His-tagged Csy3, pTRB30 derivative, Km^R^	[20]
pJSC6	N-term His-tagged Cas6f, pTRB30 derivative, Km^R^	[20]
pJSC9	Native Cas3, pTRB30 derivative, Km^R^	This study
pJSC10	N-term His-tagged Cas1, native Cas3, pTRB30 derivative, Km^R^	This study
pJSC11	Native Csy1-3, Cas6f, pBAD30 derivative, Ap^R^	This study
pMAT4	N-term His-tagged SdhE, pTRB30 derivative, Km^R^	M. McNeil; unpublished
pTG116	N-term FLAG-tagged Cas6f, pBAD30 derivative, Ap^R^	[20]
pTG117	C-term FLAG-tagged Cas6f, pBAD30 derivative, Ap^R^	[20]
pTG126	N-term FLAG-tagged Csy1, pBAD30 derivative, Ap^R^	This study
pTG127	C-term FLAG-tagged Csy1, pBAD30 derivative, Ap^R^	This study
pTRB30	pQE-80L (Qiagen) based expression vector, Ap^R^ replaced by Km^R^	[20]

**Table 2:** Oligonucleotides used in this study.

Name	Sequence (5′-3′)	Description	Restriction site
CR21	CGTCTAGATTAGTAATCGAATTCGTAGGAAGTGTC	R *csy2*	XbaI
CR23	GCTCTAGACTTGTCATCGTCGTCCTTGTAGTCGTAATCGAATTCGTAGGAAGTGTC	R *csy2*, C- FLAG-tag	XbaI
CR25	GCTCTAGATTATTCGCCTTTTTCACCAAACACACCG	R *csy3*	XbaI
CR27	GCTCTAGATTACTTGTCATCGTCGTCCTTGTAGTCTTCGCCTTTTTCACCAAACACACCG	R *csy3*, C- FLAG-tag	XbaI
CR28	CGGAGCTCAAGAGGAGAAATTAACTATGGACTACAAGGACGATGACGATAAGAGCACGTTGATTATCCTCCGCCG	F *csy2*, N- FLAG-tag	SacI
CR29	CGGAGCTCAAGAGGAGAAATTAACTATGAGCACGTTGATTATCCTCCGCCG	F *csy2*	SacI
CR30	CGGAGCTCAAGAGGAGAAATTAACTATGGACTACAAGGACGATGACGATAAGGCAAAAGCAGCAACGACGTTG	F *csy3*, N-FLAG-tag	SacI
CR31	CGGAGCTCAAGAGGAGAAATTAACTATGGCAAAAGCAGCAACGACGTTG	F *csy3*	SacI
JCO2	TTTAAGCTTTCAACTGAGTGCGCCAAACAC	R *cas3*	HindIII
JCO5	ATAGCATGCTTAGAACCACGGAACGGTG	R *cas6f*	SphI
PF138	CACACTTTGCTATGCCATAG	F MCS pBAD30	
PF139	GCTACTGCCGCCAGG	R MCS pBAD30	
PF209	TCGTCTTCACCTCGAGAAATC	F MCS pTRB30	
PF210	GTCATTACTGGATCTATCAACAGG	R MCS pTRB30	
PF281	TTTGAATTCAGGAGAAATTAACTATGAACATTCTGCTGATTTC	F *cas3*	EcoRI
TGO34	AGGTGGATCCATGGATAACGCCTTTAGCC	F *cas1*	BamHI
TGO37	AGGTCTGCAGCGCACTCAACTGAGTGC	R *cas3*	PstI
TGO58	TACCCGGGAAGAGGAGAAATTAACTATGGACTACAAGGACGATGACGATAAGATGAGAAATGGACTACCCG	F *csy1*, N-FLAG-tag	XmaI
TGO59	ATAAAGCTTTCAACGTGCTCATGCCAG	R *csy1*	HindIII
TGO60	TACCCGGGAAGAGGAGAAATTAACTATGAGAAATGGACTACCCG	F *csy1*	XmaI
TGO61	ATAAAGCTTTTACTTATCGTCATCGTCCTTGTAGTCTGCCAGCTCCTCTTTCAG	R *csy1*, C-FLAG-tag	HindIII

### Construction of His-tagged Cas and Csy Expression Vectors

Expression vectors, encoding the *P. atrosepticum* Cas1, Cas3, Csy1-3 and Cas6f proteins carrying N-terminal hexahistidine extensions (MRGSHHHHHHGS), were constructed previously [20]. A construct for the expression of N-terminally His-tagged Cas1 and native Cas3 (pJSC10) was generated by amplifying the *cas1-cas3* region with primers TGO34 and TGO37. The resulting 4.3 kb PCR product was digested with BamHI and PstI and ligated into pTRB30, previously cut with the same enzymes. Primers PF209 and PF210 flank the pTRB30 MCS and were used for sequencing all pTRB30 derivatives, in combination with internal gene-specific primers.

### Construction of FLAG-tagged Csy and Cas6f Expression Vectors

FLAG-tagged Csy vectors were generated as follows: the *csy1* gene (1332 bp) was amplified by PCR using primers TGO58 and TGO59, for generation of an N-terminal FLAG-tag, or TGO60 and TGO61, for generation of a C-terminal FLAG-tag. The *csy2* gene (933 bp) was amplified by PCR using primer pairs CR28 and CR21, for generation of an N-terminal FLAG-tag, or CR29 and CR23, for generation of a C-terminal FLAG-tag. The *csy3* gene (1014 bp) was amplified by PCR using primer pairs CR30 and CR25, for generation of an N-terminal FLAG-tag, or CR31 and CR27, for generation of a C-terminal FLAG-tag. The *csy1* products were digested with XmaI and HindIII, the *csy2* and *csy3* products digested with SacI and XbaI and all were cloned into pBAD30, previously cut with the same enzymes. N- and C-terminally FLAG-tagged Cas6f vectors were constructed previously [20]. Primers PF138 and PF139 flank the pBAD30 MCS and were used for sequencing all pBAD30 derivatives, in combination with internal gene-specific primers.

### Construction of Native Expression Vectors

A construct for the expression of Csy1-3 and Cas6f (pJSC11) was generated by amplifying *csy1-3, cas6f* with primers TGO60 and JCO5. The resulting 3.8 kb PCR product was digested with XmaI and SphI and ligated into pBAD30, previously cut with the same enzymes. A plasmid that expressed native Cas3 (pJSC9) was constructed by amplifying the *cas3* gene with primers PF281 and JCO2, digesting the product with EcoRI and HindIII and ligating into EcoRI/HindIII-digested pTRB30.

### Co-affinity Purification

*P. atrosepticum* Δ*cas* (PCF80) cell cultures (500 ml) carrying the *csy1-3* and *cas6f* genes (pJSC11) and one other *cas* or *csy* gene (on pTRB30-derived plasmids) were induced with 1 mM IPTG and 0.1% arabinose at an OD_600_ of 0.5 and grown for further 20 hours. As a control, an unrelated protein (His-SdhE [40]) was expressed from pTRB30 (pMAT4) with Csy1-3 and Cas6f co-expressed from pJSC11. Co-purification experiments of His-Cas1 and Cas3 were performed identically except plasmid pJSC10 (His-Cas1, Cas3) was used in the presence or absence of pJSC11 (Csy1-3, Cas6f). Cells were pelleted by centrifugation at 3030×*g* for 15 min, the supernatant removed and the cell pellet frozen at −20°C overnight. To enable cell lysis, pellets were resuspended in 5 ml of 50 mM NaH_2_PO_4_, 300 mM NaCl, 10 mM imidazole, 0.2 mg/ml lysozyme (Roche) and protease inhibitor cocktail (Sigma) and incubated for 30 min on ice. Following sonication, insoluble material was removed by centrifugation for 30 min at 12100×*g* at 4°C and the supernatant containing the soluble proteins was loaded on Ni-NTA (Qiagen). Unbound protein was removed by washing with 20–30 column volumes of 50 mM NaH_2_PO_4_, 300 mM NaCl, 40 mM imidazole. Proteins that had specifically bound were eluted with 50 mM NaH_2_PO_4_, 300 mM NaCl, 250 mM imidazole. Where applicable protein was dialyzed into 20 mM HEPES, 300 mM KCl, 5% Glycerol, 1 mM DTT and further purified by size exclusion using a Superose 12 (10/300) GL column (GE Healthcare). Fractions were analysed by SDS-PAGE and Coomassie staining. For SDS-PAGE, proteins were separated on 12% or 15% polyacrylamide gels using the Mini-PROTEAN Tetra Cell (Biorad) and a pre-stained protein ladder (Invitrogen). For Coomassie staining, gels were fixed in 40% (v/v) 2-propanol and 10% (v/v) acetic acid and stained with 0.01% (w/v) Coomassie Brilliant Blue G-250 (Merck).

### Protein Identification by Mass Spectrometry

Excised protein bands were subjected to in-gel digestion with trypsin following previously described protocols [41]. Eluted peptides were dried using a centrifugal concentrator. Samples were re-solubilised in 5% [v/v] acetonitrile, 0.2% [v/v] formic acid in water and injected onto an Ultimate 3000 nano-flow uHPLC-System (Dionex Thermo Scientific, Co,CA) that was in-line coupled to the nanospray source of a LTQ-Orbitrap XL hybrid mass spectrometer (Thermo Scientific, San Jose, CA). Peptides were separated on an in-house packed emitter-tip column (75 um ID PicoTip fused silica tubing (New Objectives, Woburn, MA) packed with C-18 material on a length of 8–9 cm) by a gradient developed from 5% [v/v] acetonitrile, 0.2% [v/v] formic acid to 80% [v/v] acetonitrile, 0.2% [v/v] formic acid in water over 35 min at a flow rate of 400 nl/min. Full MS in a mass range between m/z 300–2000 was performed in the Orbitrap mass analyser with a resolution of 60,000 at m/z 400. The strongest 5 signals were selected for CID (collision induced dissociation)-MS/MS in the LTQ ion trap at a normalized collision energy of 35%. For protein identification, MS/MS data were searched against an in-house Mascot server (http://www.matrixscience.com). The search was set up for full tryptic peptides with a maximum of three missed cleavage sites. Carboxyamidomethyl cysteine, oxidized methionine, and pyroglutamate (E, Q) were included as variable modifications where appropriate. The precursor mass tolerance threshold was 10 ppm and the max fragment mass error was 0.8 Da. The significance of the predicted protein matches was calculated using the probability based scoring, termed the Mascot score (matrix science). The scores are calculated based on the peptide matches and the probability of these matches occurring at random. Stated simply, the higher the score, the greater the confidence of a significant match.

### Co-Immunoprecipitation and Western Blotting

In *P. atrosepticum* WT or Δ*cas* strains, either N- or C-terminally FLAG-tagged Csy or Cas6f proteins served as bait and were co-expressed with an N-terminally His-tagged Csy of Cas6f protein as prey. Co-expression of the His-Csy or His-Cas6f constructs with pBAD30 was performed as a negative control. Cell cultures (50 ml) were induced with 1 mM IPTG and 0.1% arabinose at an OD_600_ of 0.5, incubated for a further 4 h and pelleted for 15 min at 4°C and 3030×*g*. Co-IP was carried out using the FLAG® Tagged Protein Immunoprecipitation Kit (Sigma) according to the manufacturer’s instructions and as described previously [20]. Analysis of total cell extracts, wash fractions and eluted protein was performed by Western blotting as described previously [20]. Mouse monoclonal anti-His (Sigma) or anti-FLAG M2 (Sigma) were used as primary antibodies and as a secondary antibody, goat anti-mouse IgG-HRP (Santa Cruz) was used. Bands were visualized on X-Ray film (AGFA) using the SuperSignal® West Pico Chemiluminescent Substrate Kit (Pierce).

## Results and Discussion

### The Csy1-3 and Cas6f Proteins Co-purify

The formation of a complex of subtype-specific proteins has been observed across different types of CRISPR/Cas systems and plays a key role in interference [10], [11], [28], [29], [33]. We hypothesised that the *P. atrosepticum* subtype I-F specific proteins also formed a complex. To test this, an affinity co-purification and mass spectrometry approach was utilised. These experiments were performed in a Δ*cas* strain with an entire deletion of the *cas1, cas3, csy1-3, cas6f* operon. This strain still expresses the WT CRISPR1-3 arrays and generates mature crRNAs when Cas6f is complemented, while there is no interference from chromosomally expressed Cas proteins [20]. *P. atrosepticum* Δ*cas* strains were generated that each contained two plasmids, one expressing native Csy1-3 and Cas6f (pJSC11) and a second expression vector encoding a single N-terminally His-tagged Csy (Csy1-3) or Cas6f protein (pJSC3-6; all plasmids are listed in Table 1). Csy and Cas6f protein expression was induced in all four *P. atrosepticum* strains and the His-tagged Csy or Cas6f proteins were purified on Ni-NTA agarose under native conditions. Elution fractions were separated by SDS-PAGE to visualise co-purified proteins, which demonstrated proteins of the predicted masses of Csy1-3 and Cas6f (Fig. 1B). The predominant individual protein bands, and the protein content in entire lanes, were identified following trypsin digestion and mass spectrometry using an LTQ Orbitrap hybrid mass spectrometer, which enables high accuracy peptide determination in complex protein samples.

The co-purification of all Csy proteins was enriched following purification of each His-tagged Csy protein when compared with purification of an unrelated bait protein, His-SdhE (Fig. 1B and Table 3). Purification of either Csy1 or Csy2 resulted in a clear co-purification of the other (Fig. 1B and Table 3). Furthermore, Cas6f appears weakly associated with the complex when co-purified with His-tagged Csy1, Csy2 or Csy3. Indeed, the His-Csy1 or His-Csy2 baits gave only very faint bands of co-purified Cas6f on SDS-PAGE, but Cas6f was detected by MS. In contrast, when His-tagged Cas6f is used as the bait, there is a clear co-purification of the other Csy proteins (Fig. 1B and Table 3). In agreement, a study published during the preparation of our manuscript showed that in *Pseudomonas*, mature crRNAs generated by Cas6f were required for complex assembly [42]. In *Pectobacterium*, overexpression of Cas6f increased crRNA generation [20], which could explain the increased complex co-purification with higher Cas6f concentrations when also expressed from the bait plasmid. Surprisingly, in the band at ∼26 kDa, which is present in all four pulldowns, Cas6f and to a lesser extent Csy3 were identified. At this point we are unable to explain this alternative migration pattern. Overall, our results are in agreement with a recent report, which showed that the homologous Csy1-3 and Cas6f proteins from *P. aeruginosa* co-purified along with crRNA [35]. Wiedenheft *et al.* co-purified Csy1-3 and Cas6f using a heterologous *E. coli* system with over-expression of the pre-crRNA. In our study we have performed the analysis in the cognate host and utilised the physiological levels of pre-crRNA expression [35]. Taken together, these data demonstrate that the Csy1-3 and Cas6f proteins from different subtype I-F systems interact and form a complex.

**Table 3:** Co-purification of Csy1-3 and Cas6f proteins as detected by MS.

Bait	Protein	Size (aa)^a^	MW (Da)^b^	Peptides	Coverage (%)		Score^c^
Control	Csy1	443	50385	5	10	177	
(His-SdhE)	Csy2	310	34861	4	7	103	
	Csy3	337	36910	0	0	0	
	Cas6f	184	20459	0	0	0	
His-Csy1	Csy1	443	50385	288	97	9271	
	Csy2	310	34861	102	90	2893	
	Csy3	337	36910	47	52	1442	
	Cas6f	184	20459	5	35	169	
His-Csy2	Csy1	443	50385	201	96	4206	
	Csy2	310	34861	87	97	1856	
	Csy3	337	36910	12	26	394	
	Cas6f	184	20459	4	25	157	
His-Csy3	Csy1	443	50385	113	74	2074	
	Csy2	310	34861	30	43	555	
	Csy3	337	36910	114	96	4850	
	Cas6f	184	20459	6	37	193	
His-Cas6f	Csy1	443	50385	59	55	1353	
	Csy2	310	34861	20	33	428	
	Csy3	337	36910	74	76	2209	
	Cas6f	184	20459	121	97	3290	
His-Cas1	Cas1	326	36259	117	67	4256	
	Csy1	443	50385	4	10	135	
	Csy2	310	34861	2	6	99	
	Csy3	337	36910	2	6	99	
	Cas6f	184	20459	0	0	0	
His-Cas3	Cas3	1098	124893	142	40	2932	
	Csy1	443	50385	16	23	348	
	Csy2	310	34861	8	26	190	
	Csy3	337	36910	8	16	219	
	Cas6f	184	20459	3	18	122	

a Size in amino acids of the WT protein sequence.

b Theoretical average MW.

c Mowse score as determined by Mascot (Matrix Science).

### Protein-protein Architecture of the Csy1-3, Cas6f Complex

**Figure 2:**
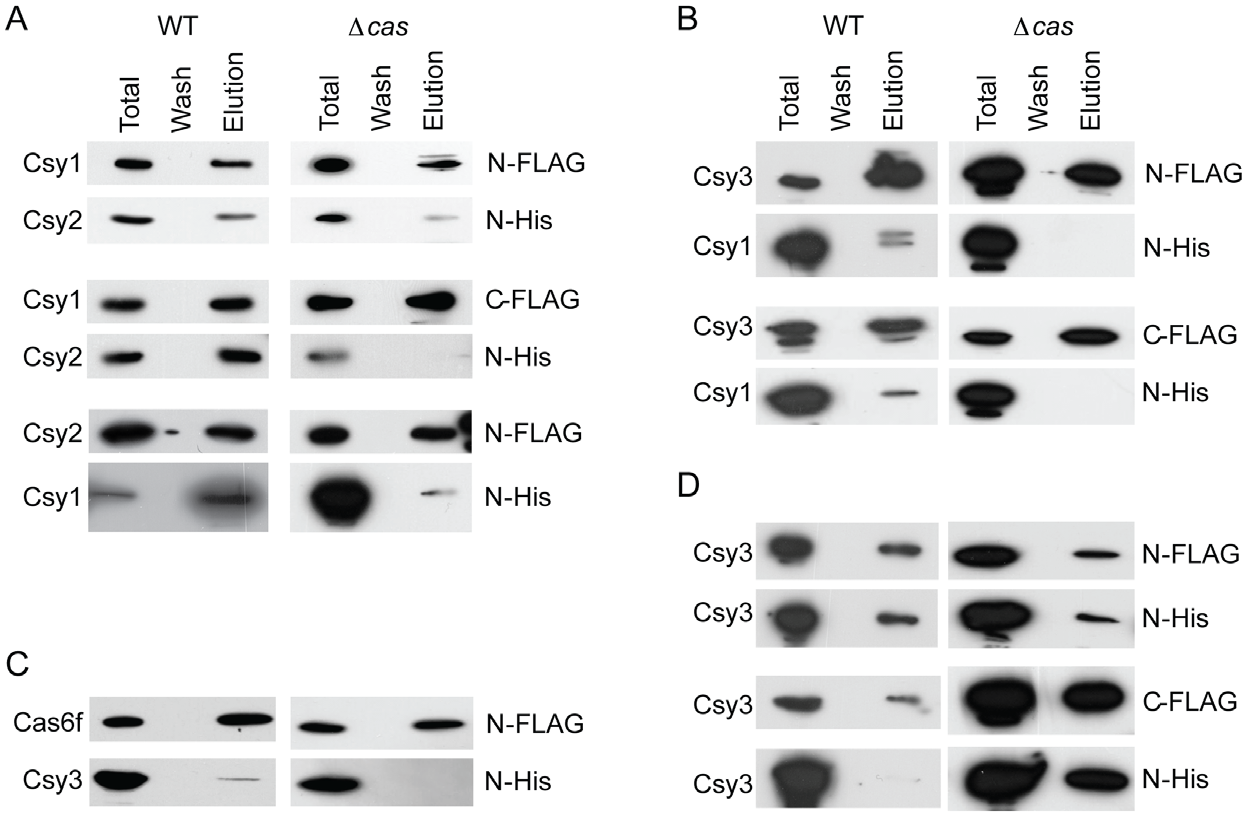
Csy1-3 and Cas6f protein-protein interactions in WT and Δ*cas* **strains.** N- or C-terminally FLAG-tagged Csy proteins were expressed in the presence of N-terminally His-tagged Csy and Cas6f proteins. Proteins were expressed, cells were lysed, proteins purified on anti-FLAG agarose, washed and eluted. Fractions were separated by SDS-PAGE and proteins were detected by Western blotting. Lanes indicate protein expression (Total), the final wash (Wash) and the elution fraction (Elution). (A) Csy1 and Csy2 interact in the absence of other Cas or Csy proteins. (B) Csy3 and Csy1 interact in the WT but not in the Δ*cas* mutant background. (C) Cas6f and Csy3 interact in the WT but not in the Δ*cas* mutant background. (D) Csy3 self-assembles.

The co-purification and MS approach described above indicated the formation of a complex composed of Csy1-3 and Cas6f. However, it was important to verify the formation of this complex using an alternative approach and to probe in more detail the individual protein-protein interactions. To achieve this, each Csy protein and Cas6f were FLAG-tagged separately at both the N- or C-terminus and each construct was used as the bait in co-immunoprecipitation (Co-IP) experiments with each prey Csy or Cas6f protein containing an N-terminal His-tag. Every possible combination of Co-IP experiments (32 in total) were performed in the WT background to determine which protein-protein interactions could be detected *in vivo* in the presence of both native crRNA production and other chromosomally-encoded Cas and Csy proteins (Fig. 2). In addition, a further complete set of 32 Co-IPs were performed in the Δ*cas* strain lacking the entire *cas1, cas3, csy1-3, cas6f* operon. As mentioned above, this strain still contains the WT CRISPR1-3 arrays, but cannot generate mature crRNAs due to the lack of Cas6f [20]. Therefore, this strain enabled an assessment of interactions which still occur *in vivo* in the absence of mature crRNAs and other native Cas/Csy proteins (Fig. 2). A summary of the results for all Co-IPs performed is presented in Table 4.

**Table 4:** Summary of Cas and Csy protein Co-IP results.

		His-Csy1		His-Csy2		His-Csy3		His-Cas6f	
	FLAG	WT	Δ*cas*	WT	Δ*cas*	WT	Δ*cas*	WT	Δ*cas*
Csy1	N-term	−a	−	+^b^	+	−	−	−	−
	C-term	−	−	+	−	−	−	−	−
Csy2^c^	N-term	+	+	−	−	−	−	−	−
	C-term	−	−	−	−	−	−	−	−
Csy3	N-term	+	−	−	−	+	+	−	−
	C-term	+	−	−	−	−	+	−	−
Cas6f	N-term	−	−	−	−	+	−	+	+
	C-term	−	−	−	−	−	−	−	−

a − no detection of His-prey with FLAG-bait.

b + detection of His-prey with FLAG-bait.

c C-term FLAG-tagged Csy2 protein was detected as truncated.

Csy1 and Csy2 co-purified in both the WT and somewhat weaker in the Δ*cas* strain, suggesting this interaction does not require other Cas or Csy proteins nor does it require crRNAs (Fig. 2A). The stronger interaction in the WT indicates that the presence of the other proteins and/or the crRNA helps to stabilize the interaction without being essential. Indeed, His-Csy1 and native Csy2 can be co-purified from the Δ*cas* mutant, the two co-elute on a size exclusion column and the stability of purified Csy2 is increased in the presence of Csy1 (J. T. Chang, C. Richter and P. C. Fineran, unpublished data). Likewise, Csy1 and Csy2 from *P. aeruginosa* could be co-purified from *E. coli* independently of the other Csy proteins [35]. When we expressed C-terminally tagged Csy2 (either FLAG- or His-tagged), this resulted in a truncated version of the protein of about 26 kDa, which did not interact with Csy1.

Csy1 and Csy3 co-purified weakly in the WT but not in the Δ*cas* background (Fig. 2B). The requirement of the WT background for the Csy1-Csy3 interaction indicated an involvement of one or all of crRNA, Csy2 or Cas6f, albeit indirectly, since no interactions between Csy1 and Cas6f or Csy3 and Csy2 were detected (Table 4). It is probable, given the coupling of Csy1 to Csy2, that a heterodimer of these proteins is required to interact with Csy3 but the crRNA is also likely to play a role.

Csy3 and Cas6f interacted very weakly in the WT background, but not in the Δ*cas* strain (Fig. 2C). As shown previously, Cas6f is sufficient to generate crRNAs [20]. Hence, we predict that Csy1 and Csy2 are also required to mediate the Csy3-Cas6f interaction. A requirement for Csy1 and Csy2 could also explain the low yield of co-purified Cas6f as the Csy3-Cas6f interaction shown in Figure 2C would depend on the lower native chromosomal expression of Csy1 and Csy2.

Finally, a strong interaction between Csy3 and itself was detected in both the WT and Δ*cas* strains, consistent with it forming a dimer or higher order multimer (Fig. 2D). In support of a multimeric Csy3, purification of His-Csy3 results in purified protein that has a tendency to aggregate and could not be resolved by size-exclusion chromatography (C. Richter and P. C. Fineran, unpublished data).

Previously, we demonstrated that Cas6f self-interacts in both WT and Δ*cas Pectobacterium* backgrounds, [20], but a Cas6f dimer was not observed in the *Pseudomonas* complex [35]. The role of the Cas6f self-interaction is unknown, but could be due to multiple Cas6f proteins bound to one pre-crRNA or a consequence of an increase in Cas6f relative to the Csy1-3 proteins due to overexpression.

**Figure 3:**
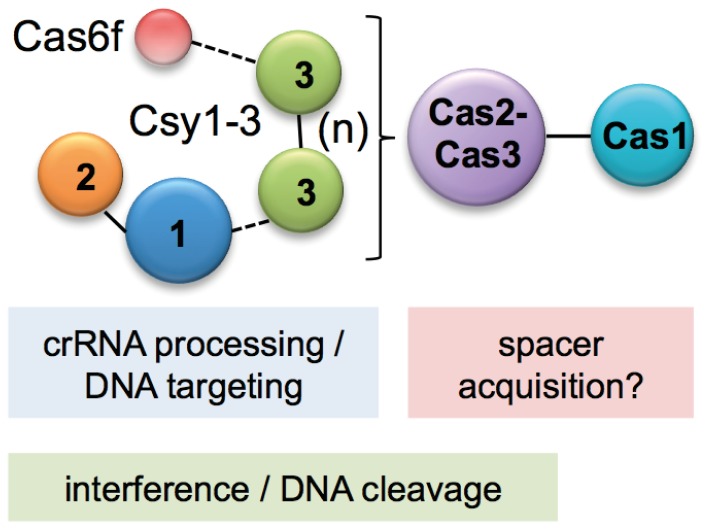
Summary of protein interactions in the CRISPR/Cas subtype I-F system. Protein interactions detected by Co-IP are shown as dashed lines (interact only in WT) or solid lines (interact in WT and Δ*cas*). The Csy3-Csy3 interaction is denoted (n) as multiple Csy3 proteins could interact. Cas3 (Cas2-Cas3 hybrid) was shown to co-purify the Csy1-3 and Cas6f proteins and also co-purify with Cas1. In the subtype I-F systems, Cas6f is involved in crRNA generation [20], [26] and Csy1-3, Cas6f bound to a crRNA can bind complementary DNA targets [35] and requires Cas3 for interference [46]. Cas3 (Cas2-Cas3) and Cas1 are predicted to be involved in spacer acquisition.

This exhaustive Co-IP approach demonstrated that the organisation of the Csy complex follows the arrangement of Csy2-Csy1-Csy3(n)-Cas6f (summarised in Figure 3), which is consistent with the observations from the complex pulldown assays (Table 3). Csy1 and Csy2 appear to form one end of the complex while a Csy3 multimer interacts with Csy1 and Cas6f, bridging the two and forming the backbone of the complex. The recent study of a similar complex from *P. aeruginosa* used native MS and size exclusion chromatography to predict that the stoichiometry was Csy1_1_:Csy2_1_:Csy3_6_:Cas6f_1_:cRNA_1_ with a MW of ∼350 kDa [35]. The same authors used TEM and small-angle X-ray scattering to identify a 120×150 Å crescent-like structure with a regular repeating feature, suggesting Csy3 forms the backbone. Our Co-IP interaction data showing that Csy3 self-interacts and forms protein-protein interactions with Csy1 and Cas6f corroborates this model and provides alternative and additional evidence for this arrangement of the subtype I-F complexes. Furthermore, our data shows for the first time that Csy3-Csy3 and Csy1-Csy2 interact *in vivo* without the requirement for other Cas or Csy proteins or mature crRNAs. In *Pseudomonas*, the Csy1-3 and Cas6f arch is 200 Å in length, consistent with a crRNA lying along the length of the complex [35]. We have demonstrated that *Pectobacterium* Cas6f processes the pre-crRNA [20] and, in *Pseudomonas*, Cas6f retains bound crRNA at the stem-loop of the repeat [26], [43]. Recently, crRNA maturation by Cas6f was shown to be necessary for Csy1-3, Cas6f-crRNA complex assembly in *Pseudomonas* [42]. Taken together with our data, we propose that following crRNA generation by Cas6f, Csy1 and Csy2 bind the 5′ 8 nt handle (hence the Csy1-Csy2 interaction does not require Csy3 or Cas6f). Next, Csy3 binds Csy1, oligomerizes and binds non-specifically to the variable crRNA spacer sequence to complete the complex via interaction with Cas6f. In this model, the location of Csy1 and Csy2 on the 5′ 8 nt handle, suggests Csy1, Csy2 and the handle would be important in distinguishing target from non-target during interference [44].

### Cas3 but not Cas1 Interacts with the Csy1-3, Cas6f Complex

Subtype-specific complex formation has been detected for the *E. coli* subtype I-E system. Cascade contains a single crRNA [28], [29] and is able to bind to target DNA in a sequence-specific manner [28] but requires the presence of Cas3 to mediate the inhibition of phage infection via cleavage of the target DNA [10], [38]. Cas3 is the signature protein of type I CRISPR/Cas systems, containing nuclease and helicase domains [14] and aids interference by unwinding and cleaving the target DNA [36]–[38], [45].

We hypothesised that the subtype I-F Cas3 would interact with the Csy complex. Co-IPs were performed with each of N- or C- terminal FLAG-tagged Csy1-3 and Cas6f as bait and N-terminal His-Cas3 as the prey in WT and Δ*cas P. atrosepticum* but no interactions were detected (data not shown). In a complementary and non-directed approach, His-Cas3 was expressed in the presence of the Csy1-3 and Cas6f proteins in the Δ*cas* strain and purified under native conditions. The elution fractions were analysed by SDS-PAGE and co-purifying proteins identified using a highly-sensitive LTQ Orbitrap hybrid MS. Csy1, Csy2, Csy3 and to a lesser extent Cas6f, were all enriched upon co-purification with Cas3 compared with an unrelated control protein (Table 3). This result suggested that Cas3 can interact with the Csy complex. Interestingly, Westra *et al.* recently showed that the *E. coli* subtype I-E Cascade binds the complementary target DNA and then recruits Cas3 [38]. Therefore, it is likely that the Cas3-Csy complex interaction also requires the presence of a target DNA sequence. In our experiments target DNA was not supplied exogenously, which might explain the lower protein coverage and score for co-purification of Csy1-3 and Cas6f with Cas3 when compared with the other Csy protein baits (Table 3). However, a crRNA generated from CRISPR2 contains a spacer that deviates from the consensus PAM, but has 100% identity to *eca0560* in a genomic island of *P. atrosepticum* [20]. The presence of this native crRNA:target combination might be sufficient to detect co-purification in the larger scale pull-down assays, but not for small scale Co-IP experiments. It is also possible that the PAM deviation might still allow recruitment of Cas3 but result in a lower affinity interaction with the Csy complex [22].

An identical experiment was performed using His-Cas1 as bait. However, when compared with the controls His-Cas1 did not co-purify Csy1-3 and Cas6f proteins when assessed by SDS-PAGE or LTQ Orbitrap hybrid MS analysis (Table 3), consistent with evidence that Cas1 is not required for interference by subtype I-F [46] and I-E [10] CRISPR/Cas systems. In summary, Csy1-3 and Cas6f co-purified with Cas3 but Cas1 alone did not interact with the Csy complex.

### Cas1 and Cas3 Interact

The least well characterised phase of CRISPR/Cas immunity is the acquisition of new spacer DNA from foreign genetic elements. This adaptation stage, which has been considered the highly conserved ‘information processing subsystem’ [14], involves the Cas1 and Cas2 proteins [17], [22]. In agreement, Cas1 and Cas2 are not required for interference [10]. Cas1 possesses metal-dependent endonuclease activity against dsDNA and generates ∼80 bp fragments [47], whereas different Cas2 proteins were shown to cleave single stranded RNA at U-rich regions [48] or double stranded DNA [49].

**Figure 4:**
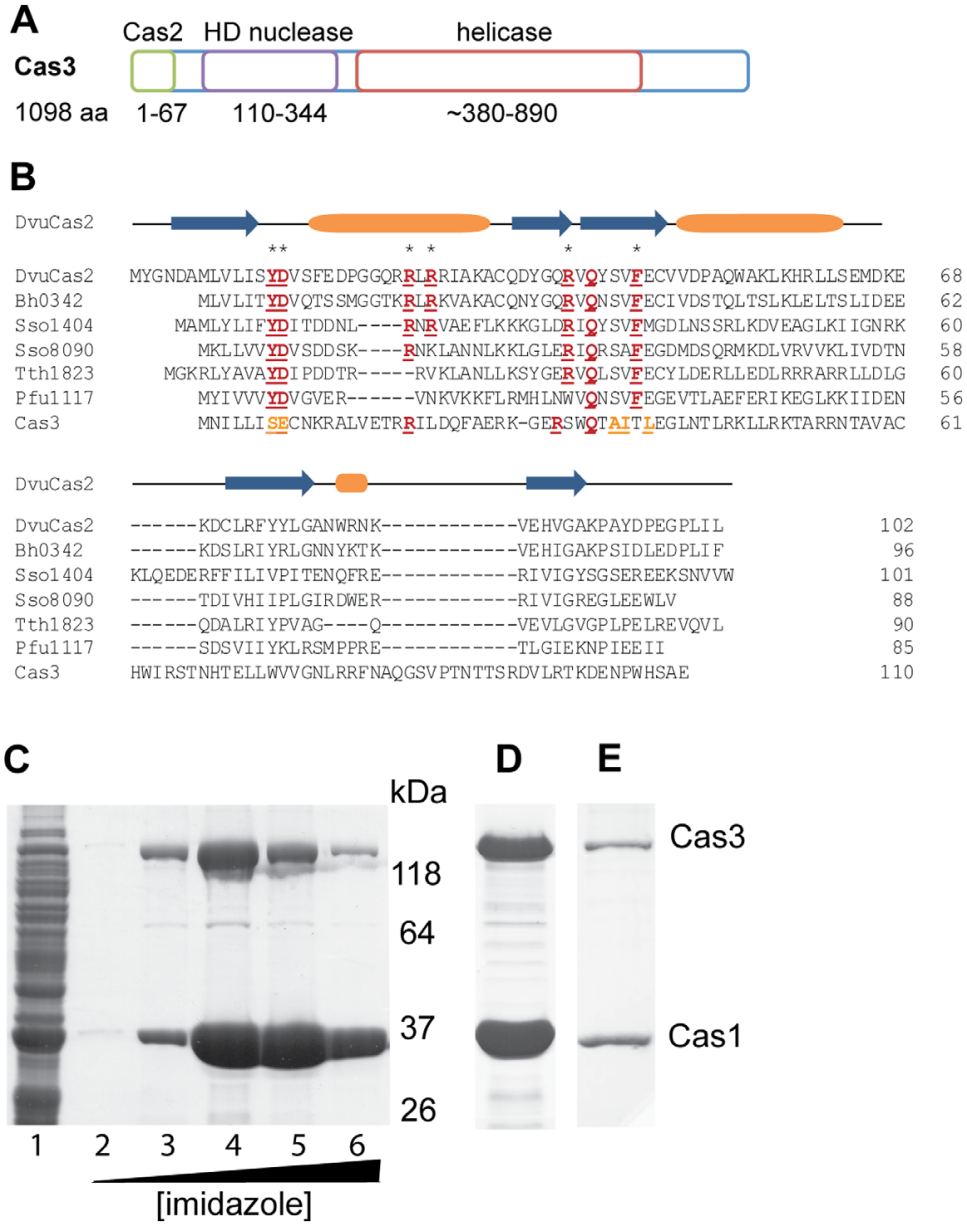
Cas1 and Cas3 interact. (A) Predicted Cas2, HD nuclease and helicase domains present in *P. atrosepticum* Cas3 based on structural homology using Phyre2 [50]. (B) Secondary structure of *Desulfovibrio vulgaris* (DvuCas2) and multiple sequence alignment of the N-terminal 110 aa of Cas3 with Cas2 homologues for which there is structural data. Blue arrows indicate β–sheets and orange barrels α–helices. Residues identified to be involved in protein function are marked with asterisks. Conserved residues are depicted in red, functionally similar residues in yellow. (C) Co-purification of His-Cas1 and Cas3 following expression in the Δ*cas* mutant (PCF80, pJSC10). Proteins in the soluble fraction (lane 1) were loaded onto Ni-NTA-agarose and washed with 40 mM imidazole. Proteins bound specifically were eluted with an imidazole gradient: 62.5 mM (lane 2), 125 mM (lane 3), 187.5 mM (lane 4) and 250 mM (lanes 5 and 6). (D) Co-purification of His-Cas1 and Cas3 in the presence of Csy1-3 and Cas6f following expression in the Δ*cas* mutant with pJSC10 (Cas1,3) and pJSC11 (Csy1-3, Cas6f). (E) Gel filtration fraction of His-Cas1 and Cas3 following an initial Ni-NTA purification. All samples were separated by SDS-PAGE and proteins visualized by Coomassie staining.

Subtype I-F CRISPR/Cas systems do not have a *cas2* gene, but their Cas3 proteins are proposed to have an N-terminal domain with homology to Cas2 (COG1343). Hence, this gene has recently been termed *cas2-cas3* [7], [14]. However, this is controversial as other groups have failed to detect a Cas2 domain in the N-terminus of the subtype I-F Cas3 from *Pseudomonas aeruginosa* [46]. We performed an analysis using a structural homology search using Phyre2 (Fig. 4A) [50].

First we searched with the full-length *P. atrosepticum* Cas3 sequence (1098 aa), which demonstrated that Cas3 matched the HD nuclease domain of both THB187 (Cas3) from *Thermus thermophilus* (24% id for amino acids 110–344) [45] and MJ0384 (Cas3) from *Methanocaldococcus jannaschii* (24% id for amino acids 110–225) [36]. The *P. atrosepticum* sequence from ∼380–890 aa matched many ATP-dependent DNA helicases. Next, we used the N-terminal 110 aa and searched again using the intensive mode, which resulted in the 4 top hits matching to Cas2 homologues from *Pyrococcus furiosus* (Pfu1117) with 16% identity and 77.6% confidence for amino acids 1–73, *Bacillus halodurans* (Bh0342) [49] with 11% identity and 55.1% confidence for amino acids 1–72, *Desulfovibrio vulgaris* (DvuCas2) [51] with 10% identity and 49.8% confidence for amino acids 1–67 and *Thermus thermophilus* (Tth1823) with 13% identity and 44% confidence for amino acids 1–67.

To further investigate the degree of homology we performed a multiple sequence alignment of the N-terminal 110 aa of Cas3 with the four Cas2 homologues obtained in the Phyre2 search and two additional proteins from *Sulfolobus*, Sso1404 [48] and Sso8090, using T-Coffee (Fig. 4B) [52]. Previously identified conserved residues with implications for protein function are D8 or D10, which coordinate a divalent metal ion in Bh0342 or Sso1404 homodimers, respectively [48], [49], and Y9, R17, R18, R31 and F37 in Sso1404 [48]. Furthermore, Q33 is highly conserved. All of these amino acids are located in the N-terminal half of Cas2, while the C-terminus is less conserved [51]. In the *P. atrosepticum* Cas2 domain, Y9 is replaced by a serine, which is shorter, but also contains an OH group on the side chain. D8/D10 is conservatively substituted by glutamic acid, which has the same charge. Residues R17 and Q33 are conserved in the *P*. *atrosepticum* Cas2-Cas3 and R31 is present in a slightly altered position. R19 is not present but does not seem to be highly conserved amongst the Cas2 homologues. In *P*. *atrosepticum*, F37 is substituted by threonine, but it is possible that a cluster of hydrophobic amino acids (A35, I36, L38) in the vicinity compensate. In summary, residues that are conserved across Cas2 proteins are also present or replaced by functionally similar amino acids in *P. atrosepticum* Cas2-Cas3. Taken together, subtype I-F Cas3 proteins contain, in addition to the nuclease and helicase domains, a Cas2-like domain at the N-terminus of the protein, which might be required for spacer acquisition (Fig. 4A and B) [7], [14].

We hypothesised that if Cas1 and Cas2 are involved in acquisition via the ‘information processing subsystem’ that these proteins might interact as an additional Cas protein complex. Hence we would also expect interaction of Cas1 with the Cas2-Cas3 fusion in the subtype I-F CRISPR/Cas system in *P. atrosepticum*. Purification of His-Cas1 under native conditions in the presence of untagged Cas3 in the Δ*cas* background led to a clear co-purification of both proteins that was confirmed by MS (Fig. 4C). Native Cas3 did not bind non-specifically to the Ni-NTA in the absence of His-Cas1 (data not shown). Furthermore, the presence or absence of the Csy1–3 or Cas6f proteins had no discernible effect on this interaction (compare Fig. 4C and 4D). The His-Cas1 and Cas3 that were co-purified were analysed by size exclusion chromatography, which demonstrated a stable His-Cas1-Cas3 complex (Fig. 4E). Taken together, these results demonstrated that Cas1 and Cas3 interact, without a requirement for crRNA or Csy1-3 and Cas6f proteins (summarised in Fig. 3). We propose that the Cas1-Cas3 complex is involved in the acquisition of new spacers in the subtype I-F system. Since in *E. coli* (type I-E) Cas1 and Cas2 are required for the integration of new spacers [17], [22], we predict that Cas2-like domain in the *P. atrosepticum* Cas3 mediates the interaction with Cas1. In agreement, an N-terminal His-tag on Cas3 interferes with the Cas1-Cas3 interaction in Co-IP experiments (data not shown).

Furthermore, a very recent report of a subtype I-A system provides an example of a Cas1-Cas2 fusion protein in *Thermoproteus tenax* [53]. The Cas1-Cas2 fusion protein interacts with Cas4 and Csa1 in a complex the authors’ term Cascis (CRISPR associated complex for the integration of spacers), but its role in adaptation is not clear [53]. We envisage that the Cas1-Cas3 complex is a similar Cascis-type complex that is likely to be involved in the adaptation stage of the CRISPR/Cas mechanism.

A remaining question is why are subtype I-F Cas3 proteins fused to Cas2 domains rather than having a separate Cas2? Although the reason for the Cas2-Cas3 fusion is unclear, recent studies in the subtype I-E system of *E. coli* have implicated Cas3 in addition to Cascade in spacer acquisition in a process termed ‘priming’ [16], [22]. In priming, the presence of an initial spacer ‘primes’ the CRISPR/Cas system for the acquisition of multiple spacers from the invading phage or plasmid that are derived from the same DNA strand as the initial spacer [16], [22]. In the subtype I-E system, Cas1, Cas2, Cas3 and Cascade are required for multiple acquisition events, which are proposed to enable the rapid adaptation to invading elements that have mutated to escape the initial spacers [22]. Therefore, it is plausible that the interaction of Cas1 with the fusion of Cas2-Cas3, not only aids initial acquisition (requiring Cas1 and Cas2), but also assists in the Cas3-dependent acquisition of multiple spacers.

### Conclusions

In summary, our study provides insight into the architecture of the Cascade-like Csy1-3, Cas6f complex based on *in vivo* protein-protein interaction experiments and demonstrates which protein interactions can occur *in vivo* in the native host background in the absence of crRNA and/or other Cas proteins. We also provide the first experimental evidence for a potential role of subtype I-F Cas3 as a double agent, being able to interact with both the Csy complex and Cas1 and thus, likely to be involved in both stages of CRISPR immunity: spacer acquisition and interference.
